# Student satisfaction-oriented physical education teacher education curriculum: A framework for evaluating implementation effectiveness and optimizing quality

**DOI:** 10.1371/journal.pone.0341397

**Published:** 2026-09-01

**Authors:** Taoqing Liu, Yaqin Ren

**Affiliations:** School of Physical Education, Guangzhou Sport University, Guangzhou, Guangdong, China; University of Sharjah, UNITED ARAB EMIRATES

## Abstract

**Research purpose:**

Based on Self-Determination Theory (SDT), this study systematically examines the effectiveness of Physical Education Teacher Education (PETE) curriculum from the perspective of student satisfaction. By mapping six course design attributes to three basic psychological needs, the study constructs a theoretical framework to explain satisfaction with PETE curriculum.

**Research methods:**

This study employs a quantitative cross-sectional survey design, involving a cross-sectional survey of 842 undergraduate students majoring in Physical Education and Sports Training at universities in Guangdong Province. Data were collected using a structured questionnaire with validated reliability and validity, based on a six-dimensional satisfaction evaluation framework. Descriptive statistics, one-way analysis of variance (ANOVA), and Pearson correlation analysis were used to reveal satisfaction disparities among different professional groups and identify key influencing factors.

**Research results:**

Descriptive statistics showed moderate overall satisfaction (M = 3.77, SD = 1.034). ANOVA revealed significant between-major differences, with Physical Education majors reporting higher satisfaction in curriculum objectives, knowledge acquisition, and competency development (p < 0.05). Chi-square test further demonstrated that Sports Training majors were 1.6 times lower willingness to pursue teaching careers intentions compared to Physical Education majors (χ² = 24.918, p = 0.002). Pearson correlation analysis confirmed that knowledge acquisition (r = 0.808), skill development (r = 0.803), and course objectives (r = 0.801) are the three factors most strongly correlated with overall satisfaction.

**Research conclusions:**

The SDT-PETE course satisfaction framework developed in this study received partial empirical support: a sense of competence is the core driver of satisfaction; significant differences were found across major types on key dimensions, reflecting a mismatch between course objectives and career aspirations; no significant differences were observed by gender or grade level, indicating that a competence-oriented physical education culture has, to some extent, mitigated traditional gender effects. This framework needs to align with career development pathways and be refined through practical integration and student feedback.

## 1. Introduction

As the core vehicle for cultivating professional physical education teachers, Physical Education Teacher Education (PETE) curriculum refers to a series of planned and organized educational and teaching content system [1]. Its quality directly impacts the professional competence of future physical education teachers and the development of lifelong physical literacy among adolescents. This program typically consists of modular components such as professional theory, motor skills, pedagogical training, and teaching practicum [2]. Aimed at transforming knowledge of physical education into transferable teaching skills [3]. The implementation of the PETE curriculum must balance knowledge, skills, and competencies to support future physical education teachers in fulfilling their three primary responsibilities: teaching, coaching, and health promotion [4]. In recent years, although the importance of physical education in the school curriculum has been widely recognized, concerns about the effectiveness of the PETE curriculum persist. These concerns stem from a clear disconnect between the curriculum's theoretical foundation and the practical needs of today's educational environment [5]. There are ongoing calls worldwide for reform of physical education curricula. However, most existing research focuses on teaching effectiveness and skill acquisition within Western educational systems [6], and there remains a lack of systematic research on how sociocultural factors influence student satisfaction in non-Western contexts [7,8]. At the same time, technological advances, diversifying social expectations, and an increasingly diverse student body have made the disconnect between theory and practice in PETE curricula increasingly apparent [9,10]. This disconnect undermines pre-service teachers’ confidence in their teaching abilities, affects their perception of the value of physical education teacher training [11], and triggers a crisis of professional identity [12,13].

Students’ satisfaction as the direct recipients of the PETE curriculum is an important indicator of curriculum-implementation effectiveness [14]. This is because the training of physical education teachers involves not only the imparting of knowledge and skills, but also the shaping of emotions, attitudes, and values [15]. However, existing research still has the following shortcomings. First, most studies adopt a competency-based framework, prioritizing quantifiable outcomes such as skill mastery and teaching effectiveness while neglecting students’ emotional experiences and the social relevance of the curriculum [16,17]. Second, existing research lacks cultural sensitivity. Research on Physical Education Teacher Education (PETE) programs in the West often implicitly assumes “gender role convergence” [18,19], suggesting that female physical education teachers face a double standard: they must demonstrate athletic ability while conforming to traditional gender norms [20]. However, this assumption may not hold true in China's competence-oriented physical education culture [21], reflecting the critical moderating role of cultural scripts. Third, most existing studies focus on satisfaction at a specific point in time and lack tracking of the program's long-term impact. Salvador-Garcia et al. similarly point out that, although research on service-learning in Physical Education (PETE) is quite extensive, long-term studies remain scarce [22]. These limitations regarding the temporal dimension necessitate the adoption of continuous feedback and adaptive learning in the curriculum.

This study innovatively incorporates academic specializations as moderating variables, revealing that goal misalignment exacerbates the systemic marginalization of students in sports training programs. A mismatch between course objectives and career aspirations intensifies role stress, which is closely associated with anxiety levels and dropout rates [23]. In the field of Physical Education and Teacher Education (PETE), this stress stems from cognitive dissonance: when theoretical training fails to equip students to meet actual teaching demands, it breeds a perception of inadequacy and career uncertainty [23]. Therefore, PETE curricula urgently require ongoing evaluation and improvement to maintain their relevance and effectiveness [24]. To address these shortcomings, this study is grounded in self-determination theory. It examines how the six design attributes of the PETE course satisfaction framework—course objectives, course structure, course content, evaluation and assessment, knowledge acquisition, and skill development—meet students’ three basic psychological needs (autonomy, competence, and relatedness), thereby influencing their overall course satisfaction. At the same time, cross-cultural research indicates that these needs predict learning satisfaction across different cultural contexts, though the pathways and relative weights may vary by culture. A qualitative study by Kaur et al. (2025) on Chinese students suggests that while the three basic psychological needs are universally relevant, their interpretation and fulfillment are uniquely influenced by the Chinese cultural context [25]. These findings suggest that when applying SDT to Chinese PETE curricula, it is necessary to balance the universality of needs with the particularities of the cultural context. Based on this, the present study poses the following research questions:

1 What is the overall level of student satisfaction with the courses? What is the relationship between the various course dimensions and overall satisfaction?
2 Examine differences in satisfaction across the various dimensions and overall satisfaction by major, as well as differences in willingness to pursue a teaching career;
3 Test whether there are significant differences in course satisfaction based on gender and grade level.

## 2. Theoretical foundation and research framework

### 2.1 The applicability of SDT theory in the PETE curriculum

Self-Determination Theory (SDT) provides a systematic theoretical framework for understanding student motivation and has had a profound impact on both classroom practice and educational policy [26,27]. The core proposition of this theory states that the fulfillment of three basic psychological needs—autonomy, competence, and relatedness—is a key prerequisite for fostering intrinsic motivation and positive developmental outcomes in individuals [28]. SDT constructs a motivational continuum ranging from amotivation to extrinsic motivation and on to intrinsic motivation. It emphasizes that individuals gradually internalize extrinsic values through the fulfillment of psychological needs, ultimately leading to behavior driven by intrinsic factors [29,30]. In educational settings, when students perceive that their learning environment meets their three basic psychological needs, their motivation to learn will exhibit a higher level of autonomy, accompanied by better academic performance and psychological adjustment [31].

The reason SDT is applicable to research on PETE curricula stems from the deep alignment between the essential characteristics of PETE curricula and the core propositions of SDT theory. PETE curricula are not only a vehicle for imparting knowledge and skills in physical education but also bear the responsibility of training pre-service teachers to become professional educators equipped with teaching competencies, professional identity, and social-emotional competencies. This process—which involves the internalization of knowledge, the acquisition of skills, and the construction of identity—corresponds precisely to the core tenets of SDT: how individuals, within specific social contexts, achieve the internalization of motivation and autonomous development through the fulfillment of psychological needs. Specifically, a sense of competence is formed through the systematic transmission of professional theoretical knowledge and the training of teaching skills [32,33], and is directly related to professional self-confidence and teaching readiness [34]. When students confirm through their coursework that they are capable of future teaching, their need for a sense of competence is met, leading to increased learning motivation and satisfaction. Autonomy is reflected in the alignment of course objectives with personal career aspirations, as well as in the freedom to choose elective courses, internship tracks, and independent topics in project-based learning [35]. When students perceive that the curriculum design aligns with their own career aspirations, their learning behaviors stem from internal motivation rather than external compulsion, thereby satisfying their need for autonomy. A sense of belonging arises from the quality of teacher-student interactions, the atmosphere of peer collaboration, and the fairness and supportiveness of assessment methods [36]; when students feel respected and accepted, they are more likely to internalize the values of the teaching profession [37].

### 2.2 Cultural context and considerations for the localization of SDT

Although self-determination theory has been widely validated in Western educational systems, a growing body of cross-cultural research suggests that the pathways to need fulfillment, their relative weights, and the strength of their relationships with outcome variables may vary across cultural contexts. In the PETE context of this study, this manifests as three key theoretical predictions. First, the need for competence may carry greater predictive weight. In Western individualistic educational contexts, satisfaction is typically associated with intrinsic motivation and positive emotional experiences, emphasizing the enjoyment of the learning process and the fulfillment derived from autonomous choice [38]. However, in the Chinese cultural context, where collectivism and instrumental rationality intertwine, students’ evaluations of satisfaction with educational services often place greater emphasis on the usefulness of the curriculum—that is, whether the course content helps achieve career goals and possesses clear employment value [20]. it can be anticipated that in the PETE course, the “sense of competence” dimension—corresponding to knowledge acquisition and competency development—may have a significantly higher predictive power for overall satisfaction than the “autonomy” dimension—corresponding to course objectives and structure—and the “sense of belonging” dimension—corresponding to evaluation and assessment.

Second, the moderating effect of major type stems from the differentiated fulfillment of autonomy needs. In 2025, Guangdong Province fully implemented a policy requiring primary and secondary school students to engage in two hours of daily physical activity on campus and attend one physical education class per day [39], while the scale of physical education teacher recruitment across various regions expanded significantly. The clarification of employment prospects helps satisfy students’ need for a sense of competence and strengthens the pathway linking academic majors to careers [40]. In the context of Physical Education and Training Education (PETE), the differences in career aspirations implied by program types may lead to varying degrees of reliance on students’ need for autonomy [41]. The Physical Education major focuses primarily on primary and secondary school physical education teachers, with career goals closely aligned with the curriculum design, whereas the Sports Training major offers multiple career paths, including coaches, sports administrators, and sports entrepreneurs. Consequently, it can be anticipated that students in sports training programs will report significantly lower course satisfaction and a significantly lower willingness to pursue teaching careers compared to students in physical education programs.

Third, gender differences may be attenuated in the Chinese PETE context [42]. Although the Chinese physical education environment emphasizes a competency-based approach, thereby weakening the direct constraints of traditional gender norms, cross-cultural research suggests that gender may indirectly moderate the relationship between a sense of belonging and course satisfaction by influencing patterns of peer interaction and perceptions of evaluative fairness [43]. The aforementioned theoretical literature, combined with educational practices in Guangdong Province, provides the theoretical foundation for the exploratory hypotheses regarding program types (H2a, H2b) and gender differences (H3a) in this study.

### 2.3 Conceptual model and assumptions

Based on the core principles of Self-Determination Theory (SDT)—which posits that the fulfillment of the needs for autonomy, competence, and relatedness are key determinants of learning motivation and satisfaction—this study treats the six design attributes of the PETE curriculum (curriculum objectives, curriculum structure, curriculum content, evaluation and assessment, knowledge acquisition, and competency development) as core variables that directly predict students’ overall satisfaction with the course and their professional identity. Furthermore, given that China's physical education system includes two specialized tracks—physical education and sports training—whose career goals align differently with course design, this study treats major type as a grouping variable to examine differences in satisfaction across course dimensions. Additionally, gender and grade level are included in the analysis to test the moderating effects of demographic variables. Table 1 summarizes the theoretical origins, operational definitions.

Based on the theoretical framework outlined above and for the purposes of subsequent data analysis, this study proposes the following testable hypotheses:

H1a: Course Objectives, Course Structure, Course Content, Evaluation and Assessment, Knowledge Acquisition, and Competence Development are all positively correlated with overall course satisfaction.

H1b: Knowledge Acquisition and Competence Development, as core sources of a sense of competence, will be key predictors of overall satisfaction.

H2a: Students majoring in Physical Education will report significantly higher overall course satisfaction than students majoring in Sports Training.

H2e: Students majoring in Physical Education will express a significantly higher willingness to pursue a teaching career than students majoring in Sports Training.

H3a: There will be no significant differences in overall course satisfaction or satisfaction across its various dimensions between students of different genders.

H3b: There will be no significant differences in overall course satisfaction or satisfaction across its various dimensions between students in different academic years (junior vs. senior).

**Table 1:** Variable definitions and measurements.

Variable Type	Variable Name	Theoretical Source	Concise Theoretical Rationale	Operational Definition
**Predictor**	Course Objectives	SDT Autonomy	Goal alignment with personal aspirations satisfies the need for autonomy, ensuring behavior stems from intrinsic self-identification rather than external compulsion [35].	Students’ evaluation of objective clarity, personal relevance, and alignment with societal needs.
**Predictor**	Course Structure	SDT Autonomy + Relatedness	Flexible structures and choices provide autonomy, while coherent progression and interactive opportunities create a supportive environment for relatedness [36].	Students’ evaluation of course logic, theory-practice integration, and elective flexibility.
**Predictor**	Course Content	SDT Competence	Comprehensive, cutting-edge content relevant to practical teaching needs directly fosters a sense of competence by ensuring perceived utility and professional depth [32].	Students’ evaluation of content comprehensiveness, innovativeness, and relevance to teaching needs.
**Predictor**	Evaluation & Assessment	SDT Relatedness	Fair, diverse, and feedback-rich assessment methods demonstrate respect for individual differences, fulfilling the need for relatedness through institutional recognition [36].	Students’ evaluation of assessment variety, fairness, and recognition of efforts.
**Predictor**	Knowledge Acquisition	SDT Competence	Systematic mastery of professional theory, occupational cognition, and moral education validates cognitive readiness for the teaching profession, forming the cognitive core of competence [33].	Students’ evaluation of acquired professional, occupational, and moral education knowledge.
**Predictor**	Competence Development	SDT Competence	Practical formation of teaching skills (e.g., demonstration, instructional design) directly confirms occupational efficacy, establishing competence through actionable performance [32].	Students’ evaluation of developed teaching, demonstration, and independent instructional abilities.
**Outcome**	Overall Course Satisfaction	Comprehensive		Students’ overall satisfaction with the PETE curriculum.
**Grouping**	Major Type	Career Differentiation		Physical Education vs. Sports Training.
**Grouping**	Gender	Social Context		Male vs. Female.
**Grouping**	Grade Level	Learning Stage		Junior vs. Senior.

## 3. Materials and methods

### 3.1 Participants

The recruitment period for this study spanned from 27/12/2024 to 1/4/2025 and employed stratified cluster sampling to recruit participants from over 20 universities in Guangdong Province, China (including Guangzhou Sport University, South China Normal University, Guangdong University of Education, and Shenzhen University) offer Physical Education and Sports Training majors. Eligibility criteria required participants to be junior or senior undergraduates who had completed all required PETE curricula coursework credits. A total of 853 questionnaires were distributed electronically; after excluding 11 invalid responses due to incompleteness or logical inconsistencies, 842 valid datasets were retained, resulting in a 98.71% valid response rate. Gender distribution: Male students comprised 50.5% (n = 425) and female students 49.5% (n = 417), indicating a balanced ratio. Grade distribution: Juniors (50.2%, n = 423) and seniors (49.8%, n = 419) were nearly equally represented. Disciplinary composition: Physical Education majors (72.1%, n = 607) constituted the majority, while Sports Training majors (27.9%, n = 235), which was consistent with the actual enrollment structure of the PETE curriculum.

### 3.2 Instruments

This study used a self-administered questionnaire, *Student Satisfaction Survey on Physical Education Teacher Education curriculum Implementation*, developed through a systematic process of scale adaptation and validation. The questionnaire was designed with reference to well-established scales in the teacher education satisfaction [44,45], and localized and revised according to the specific context of the Chinese PETE curricula. The instrument comprised two sections: (1) Emographic Information: Collecting basic data such as gender, grade (junior/senior), and major (Physical Education/Sports Training); (2) Core Satisfaction Assessment: Including 33 closed-ended items organized into six dimensions: course objectives, course structure, course content, assessment and evaluation, knowledge acquisition, and competency development.

All items used a 5-point Likert scale (1 = “Strongly dissatisfied” to 5 = “Strongly satisfied”), with higher scores indicating greater satisfaction. Cronbach's alpha was used to test reliability, yielding an overall coefficient of 0.949. Subscale reliabilities for the six dimensions ranged from 0.82 to 0.96, demonstrating excellent internal consistency. Construct validity was supported by a Kaiser-Meyer-Olkin (KMO) measure of sampling adequacy of 0.958 and a significant Bartlett's Test of Sphericity (χ² = 15993.087, df = 496, p < 0.001), confirming suitability for factor analysis.To test the six-dimensional structure of the questionnaire, an exploratory factor analysis was conducted on the 33 items. As shown in Table 1, the factor loadings were 12.102 for the first factor, 3.036 for the second, 2.283 for the third, 1.865 for the fourth, 1.315 for the fifth, and 1.005 for the sixth. The items and factors in the scale accounted for 67.518% of the total variance. The rotated factor loading matrix shows that the factor loadings for these items ranged from 0.663 to 0.805, all of which were significant. Furthermore, all five identified factors met acceptable criteria. Tables 2 and 3 present the results of total variance explained, as well as the items corresponding to the extracted factors and their factor loadings, respectively.

**Table 2:** Results of the Analysis of Variance (ANOVA).

Component	Initial Eigenvalue			Sum of Squared Extraction Loads			Sum of Squared Rotational Loads		
**Total**	**Percentage of Variance**	**Cumulative %**	**Total**	**Percentage of Variance**	**Cumulative %**	**Total**	**Percentage of Variance**	**Cumulative %**
1	12.102	37.819	37.819	12.102	37.819	37.819	5.518	17.242	17.242
2	3.036	9.488	47.306	3.036	9.488	47.306	4.687	14.647	31.889
3	2.283	7.134	54.441	2.283	7.134	54.441	4.230	13.219	45.109
4	1.865	5.827	60.268	1.865	5.827	60.268	3.506	10.955	56.064
5	1.315	4.111	64.378	1.315	4.111	64.378	2.184	6.826	62.891
6	1.005	3.139	67.518	1.005	3.139	67.518	1.481	4.627	67.518
7	0.596	1.861	69.379						
8	0.576	1.801	71.180						
9	0.563	1.759	72.938						
10	0.522	1.631	74.569						
11	0.511	1.596	76.166						
12	0.474	1.481	77.647						
13	0.467	1.461	79.107						
14	0.456	1.425	80.532						
15	0.451	1.410	81.943						
16	0.424	1.326	83.269						
17	0.419	1.308	84.577						
18	0.406	1.268	85.845						
19	0.391	1.223	87.068						
20	0.386	1.208	88.276						
21	0.373	1.165	89.441						
22	0.364	1.137	90.578						
23	0.354	1.105	91.683						
24	0.347	1.083	92.766						
25	0.331	1.034	93.801						
26	0.323	1.008	94.808						
27	0.314	0.983	95.791						
28	0.292	0.911	96.702						
29	0.284	0.886	97.588						
30	0.270	0.843	98.432						
31	0.265	0.828	99.259						
32	0.237	0.741	99.897						
33	0.211	0.701	100.00						

**Table 3:** Results of the Rotated Component Matrix.

Ingredients		1	2	3	4	5	6
Knowledge Acquisition	Q19	0.704					
	Q20	0.702					
	Q21	0.663					
	Q22	0.676					
	Q23	0.748					
	Q24	0.730					
	Q25	0.713					
	Q26	0.782					
	Q27	0.787					
Course Content	Q10		0.721				
	Q11		0.779				
	Q12		0.790				
	Q13		0.770				
	Q14		0.757				
	Q15		0.752				
	Q16		0.701				
Course Structure	Q4			0.745			
	Q5			0.753			
	Q6			0.794			
	Q7			0.786			
	Q8			0.776			
	Q9			0.710			
Competence Development	Q28				0.747		
	Q29				0.805		
	Q30				0.729		
	Q31				0.73		
	Q32				0.761		
	Q33				0.654		
Course Objectives	Q1					0.780	
	Q2					0.750	
	Q3					0.778	
Evaluation & Assessment	Q17						0.784
	Q18						0.774

### 3.3 Data analysis

This study employed quantitative analysis to evaluate the implementation of PETE curricula. First, data normality was verified by the Kolmogorov-Smirnov (K-S) test (p > 0.05) to confirm that parametric test assumptions were met. Descriptive statistics were analyzed for the dimensions of course objectives and knowledge acquisition to summarize central tendency (mean) and dispersion (standard deviation). To test for between-group differences, a one-way ANOVA was used to compare satisfaction levels by academic stage (junior vs. senior) and gender (male vs. female). For the categorical variable “willingness to teach”, significant differences prompted further analysis using chi-square tests to assess associations between majors (Physical Education vs. Sports Training) and career choices. Pearson's correlation analysis explored interactions between key dimensions such as course content and assessment methods and their joint effects on overall satisfaction. To further examine the predictive power of each course dimension on overall satisfaction, a multiple linear regression analysis was conducted with overall satisfaction as the dependent variable and the six course dimensions as independent variables. All analyses were performed using IBM SPSS Statistics 27.0, with the significance level set at p < 0.05. This multi-method approach systematically identified strengths and structural variations in PETE curricula, and provided empirical evidence and rigorous countermeasures for curriculum optimization.

### 3.4 Ethical approval statement

The study “Physical Education Teacher Education Curriculum Oriented by Student Satisfaction: Implementation Effectiveness Evaluation and Quality Optimization Framework” has passed the ethical review of the Ethics Review Committee of Guangzhou Sport University; after review, it has been confirmed that this study strictly abides by the relevant ethical guidelines and norms for research involving human participants, which ensures the research process meets ethical requirements and fully protects the rights, interests and well-being of participants. The ethical approval number for this study is 2024LCLL-131, and the ethical review approval date is December 26, 2024—this approval was obtained before the official implementation of the study. The scope of approval covers the overall plan of the study, including research proposals and other supporting documents, the informed consent form, materials or advertisements for recruiting research participants, an explanation of any compensation, insurance, or benefits provided to the participants for their involvement in the study, forms and questionnaires to be completed by research participants, and the researcher's curriculum vitae and contact information.

## 4. Result

### 4.1 Descriptive statistics

**Fig 1:**
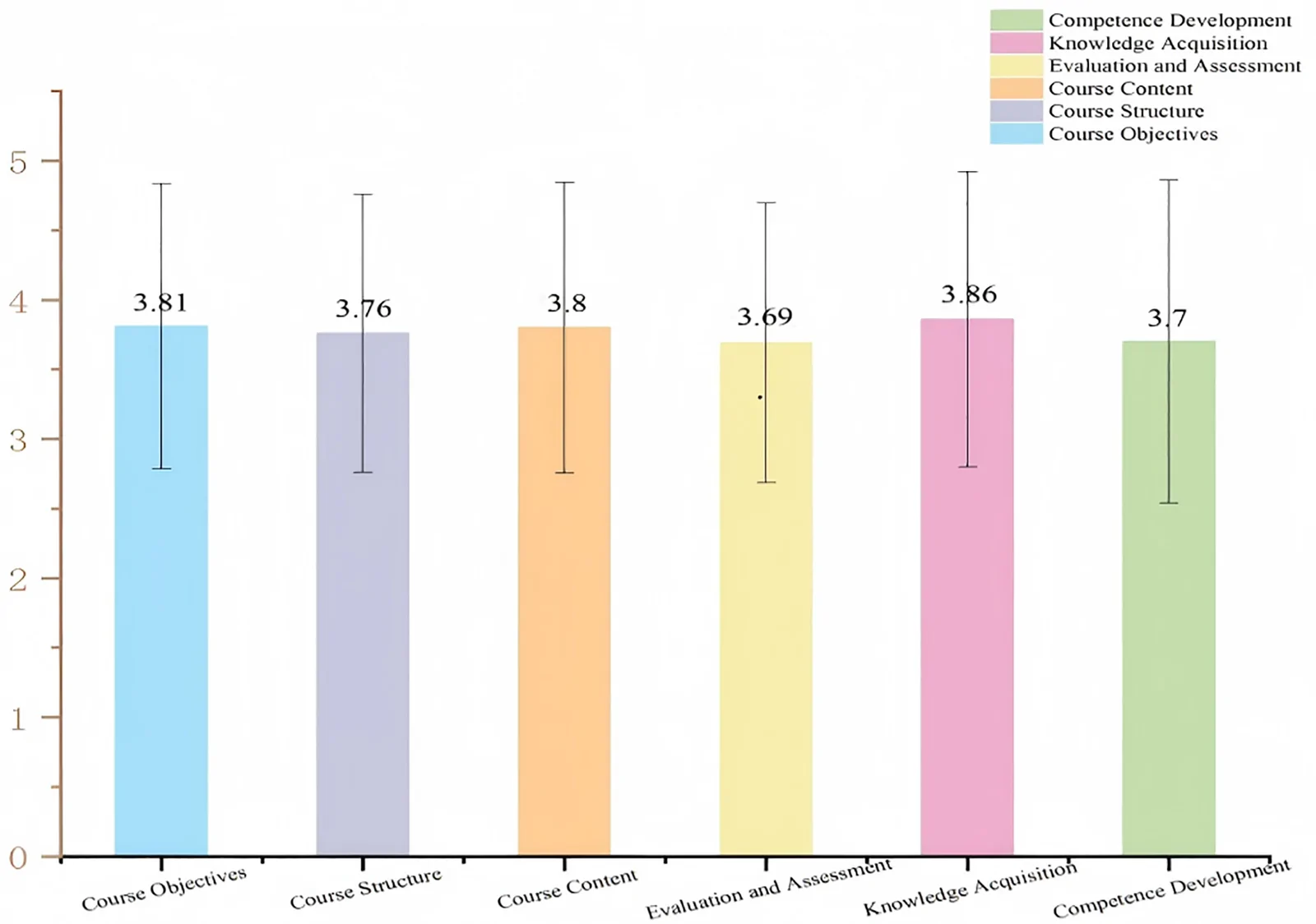
Overall Satisfaction with PETE Curriculum. Note: Knowledge Acquisition (M = 3.86) received the highest satisfaction rating, followed by Course Objectives (M = 3.81), Course Content (M = 3.80), and Course Structure (M = 3.76). Competence Development (M = 3.70) and Evaluation and Assessment (M = 3.69) received the lowest ratings.

The descriptive statistical results of the multidimensional evaluation of PETE curricula are presented in Fig 1 and Table 4. In Fig 1, shows the average satisfaction scores for the six dimensions of the PETE course, along with the corresponding standard deviations. Its six core dimensions—Course Objectives, Course Structure, Course Content, Assessment And Evaluation, Knowledge Acquisition, and Competence Development—all present significant advantages and room for improvement. Overall satisfaction across dimensions was moderate (M = 3.76, SD = 1.034). The highest satisfaction scores were found in Comprehensive course content (Q11: M = 4.06) and Career impact (Q22: M = 4.02), while the lowest scores centered on Content innovation (Q16:M = 3.48) and Practical application (Q29:M = 3.47), highlighting critical gaps in adaptive competence development.

**Table 4:** Multi-dimensional analysis of PETE curriculum.

Dimension	Key Metrics	M	SD	Strengths	Areas for Improvement
Course Objectives	Q1: Clear understanding of course objectives or expectations Q2: Course objectives are specific and operational Q3:Course objectives align with societal needs	3.81	1.025	High clarity (Q1: 3.92)	Weak societal alignment (Q2: 3.78)
Course Structure	Q4: Proportion of general teacher education courses is reasonableQ5: Proportion of physical education (PE) teacher education Q6:Proportion of practical PE courses is reasonable Q7:Course arrangements are well-connected across academic years Q8: Allocation of compulsory and elective courses is reasonable Q9:Theoretical and practical courses are well-integrated	3.76	0.998	Logical course sequence (Q7: 3.94)	Low practical ratio (Q9: 3.47)
Course Content	Q10:PETE course content is highly professional and easy to understandQ11: Physical education teaching practice course content is comprehensive and specific Q12: PETE Course content covers cutting-edge topics and hot issues Q13:PETE Course content is closely linked to actual teaching needs Q14: Course content reflects the characteristics of PE and meets professional needs Q15: Physical education teaching practice courses in various forms Q16:PETE Course content is up-to-date and innovative	3.80	1.044	comprehensive content (Q11: 4.06)	Keep up with the times(Q16: 3.48)
Evaluation & Assessment	Q17: Teachers adopt a variety of ways Q18: The usual efforts are directly proportional to the assessment results	3.69	1.005	Efforts are well rewarded (Q18: 3.71)	Lack of multiple evaluation methods(Q17: 3.54)
Knowledge Acquisition	Q19: better understand students ‘psychology and respect students’ personality differences Q20: better understand the students’ cognitive development, the characteristics of learning style and the influencing factors Q21:better understand the process of students ‘moral character and behavior habit formation, and understand the characteristics of students’ communication Q22: To have a clear understanding of the physical education teacher profession, clear the meaning of the teacher work Q23:Understand the rights and responsibilities of physical education teachers Q24:Master the theoretical knowledge of physical education teaching Q25:Master the knowledge and skills of moral education Q26:Understand the stage of the professional development of physical education teachers Q27:More willing to engage in education career	3.86	1.062	High career impact (Q22: 4.02)Clear rights and responsibilities (Q23: 4.09)	Lack of moral education module(Q25: 3.80)
Competence Development	Q28:Through the study of PETE,Mastery of PE teaching theory and practical skills Q29:Ability to independently conduct teaching Q30:Ability to moral education Q31:Ability to explain and demonstrate movements Q32:Lifelong learning ability Q33:PETE Courses are highly beneficial for future career development	3.70	1.161	Strong skill to explain and demonstrate(Q31: 3.75)	Low moral education ability(Q29: 3.55)

### 4.2 Variance analysis

A one-way ANOVA was conducted to compare course satisfaction between physical education (PE) and sports training (ST) majors(See Table 5). Results revealed significant between-group differences between the two groups on three dimensions: course objectives (F (1, 328)=6.12, p = 0.014, η² = 0.018), knowledge acquisition (F (1, 328)=5.89, p = 0.016, η² = 0.017), and Competence Development (F (1, 328)=4.97, p = 0.026, η² = 0.015). Post hoc tests indicated that physical education majors (M = 4.21, SD = 0.531) were significantly more satisfied than athletic training majors (M = 3.79, SD = 0.616) on all significant dimensions.

**Table 5:** ANOVA results by gender, grade, and major.

Factor	Dimension	F	P	Sig	η²
Gender (Male vs Female)	Course Objectives	0.446	0.504	n.s.	0.053
	Course Structure	0.098	0.754	n.s.	0.012
	Course Content	0.604	0.437	n.s.	0.027
	Evaluation and Assessment	0.147	0.702	n.s.	0.006
	Knowledge Acquisition	0.720	0.396	n.s.	0.049
	Competence Development	0.059	0.809	n.s.	0.014
Grade (Junior vs Senior)	Course Objectives	0.966	0.326	n.s.	0.031
	Course Structure	1.278	0.259	n.s.	0.035
	Course Content	1.845	0.175	n.s.	0.062
	Evaluation and Assessment	2.524	0.112	n.s.	0.055
	Knowledge Acquisition	0.144	0.704	n.s.	0.011
	Competence Development	0.810	0.368	n.s.	0.029
Major (Physical education vs Sports training)	Course Objectives	3.876	0.037	*	0.432
	Course Structure	1.153	0.283	n.s.	0.046
	Course Content	1.328	0.097	n.s.	0.031
	Evaluation and Assessment	1.624	0.203	n.s.	0.047
	Knowledge Acquisition	3.682	0.042	*	0.411
	Competence Development	3.469	0.044	*	0.429

*(Note: Significance levels: *p < 0.05, **p < 0.01, **p < 0.001; n.s. = not significant)

To explore career orientation differences, a chi-squared test of independence was performed on the categorical variable “willingness to teach” (See Table 6). A significant association emerged between major and career intentions (χ² = 24.918, p = 0.002). Notably, the percentage of ST majors unwilling to pursue teaching careers (24.7%) was 1.6 times higher than that of PE majors (15.7%),highlighting closer alignment between PETE curricula and PE students’ career trajectories.

**Table 6:** Chi-square comparison of whether different majors are willing to engage in the teaching profession.

Factor	Total	Physical Education n=607 (%)	Sports Training n=23 5(%)	χ ^2^	P
Willingness	689	512(84.3%)	177(75.3%)	24.918	0.002
Unwillingness	153	95(15.7%)	58(24.7%)		

### 4.3 Correlation analysis

Pearson correlation analyses were conducted to explore the relationships between course dimensions and overall satisfaction with the teacher education curricula. As shown in Table 7, all measured variables exhibited statistically significant positive correlations with overall satisfaction (p < 0.001). Among these, knowledge acquisition was most closely associated with overall satisfaction (r = 0.808, p < 0.001), followed by skill development (r = 0.803, p < 0.001), course objectives (r = 0.801, p < 0.001), course structure (r = 0.775, p < 0.001), course content (r = 0.760, p < 0.001), and evaluation and assessment (r = 0.703, p < 0.001). Among the correlations between variables, knowledge acquisition and skill development showed the highest correlation (r = 0.813, p < 0.001).

**Table 7:** Correlation analysis of each dimension.

variables	1	2	3	4	5	6	7
1.CO	1						
2.CS	0.796^***^	1					
3.CC	0.803^***^	0.770^***^	1				
4.EAA	0.734^***^	0.678^***^	0.713^***^	1			
5.KA	0.727^***^	0.760^***^	0.696^***^	0.750^***^	1		
6.CD	0.679^***^	0.796^***^	0.768^***^	0.779^***^	0.813^***^	1	
7.OCS	0.801^***^	0.775^***^	0.760^***^	0.703^***^	0.808^***^	0.803^***^	1

CO,course objectives;CS,course structure;CC,course content;EAA,evaluation and assessment;KA,knowledge acquisition;CD,competence development;OCS,overall course satisfaction.***p < 0.001

### 4.4 Multiple regression analysis

A stepwise multiple regression analysis was conducted with overall satisfaction as the dependent variable and the six course dimensions as independent variables(See Table 8). The results showed that the regression model was significant (F = 101.040, p < 0.001), with an adjusted R² of 0.417, indicating that the model explained 41.7% of the variance in overall satisfaction. Specifically, course objectives (B = 0.217, β = 0.185, p < 0.001), course structure (B = 0.161, β = 0.122, p < 0.001), course content (B = 0.232, β = 0.160, p < 0.001), knowledge acquisition (B = 0.309, β = 0.224, p < 0.001), and skill development (B = 0.170, β = 0.127, p = 0.007) all influence overall satisfaction and are positive predictors (positive regression coefficients). Furthermore, evaluation and assessment do not influence overall satisfaction (B = 0.0388, β = 0.224, p = 0.328), which may be related to insufficient statistical power and measurement attenuation resulting from the limited number of items in this dimension (only two items) [46]. This result further confirms that knowledge acquisition is the strongest predictor of overall satisfaction, followed by course objectives.

**Table 8:** Regression model coefficients for overall satisfaction across six dimensions.

Variables	B	β	t	p	VIF
(Constant)	−0.653		−3.589	<0.001	
CO	0.217	0.185	5.480	<0.001	1.645
CS	0.161	0.122	3.551	<0.001	1.688
CC	0.232	0.160	4.842	<0.001	1.568
EAA	0.038	0.032	0.978	0.328	1.553
KA	0.309	0.224	6.783	<0.001	1.572
CD	0.170	0.137	3.949	0.007	1.739
R^2^	0.649				
Adjusted R²	0.417				
F	101.040***				

At the same time, in statistical analyses of multicollinearity—specifically, the variance inflation factor (VIF)—a VIF greater than 10 indicates severe multicollinearity in the data. In this study, all VIF values were less than 10, indicating that multicollinearity among the independent variables was not severe.

**Fig 2:**
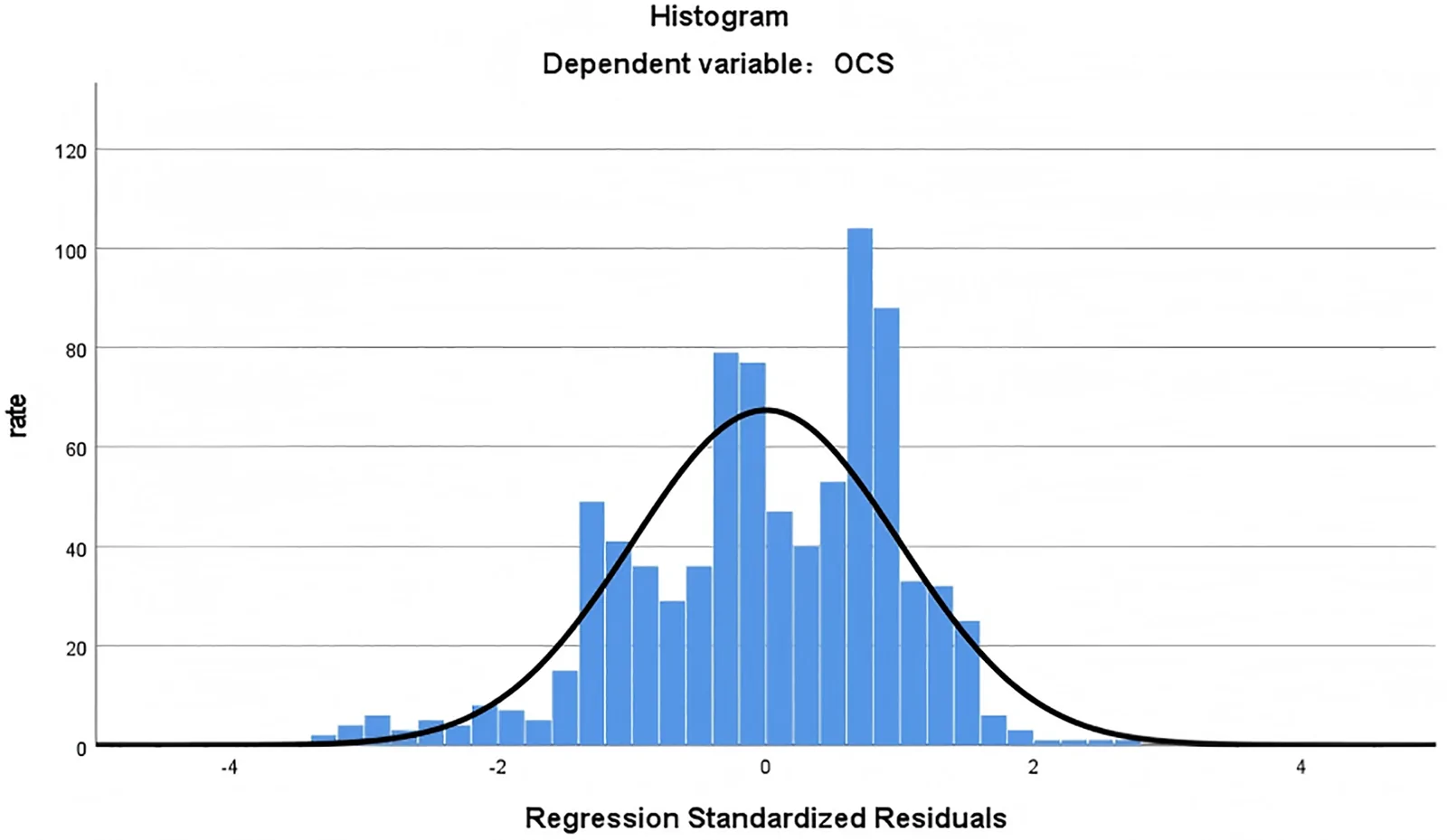
Residual Histogram. Note: The residuals exhibit a symmetric, bell-shaped distribution centered around zero, approximating a normal distribution.

**Fig 3:**
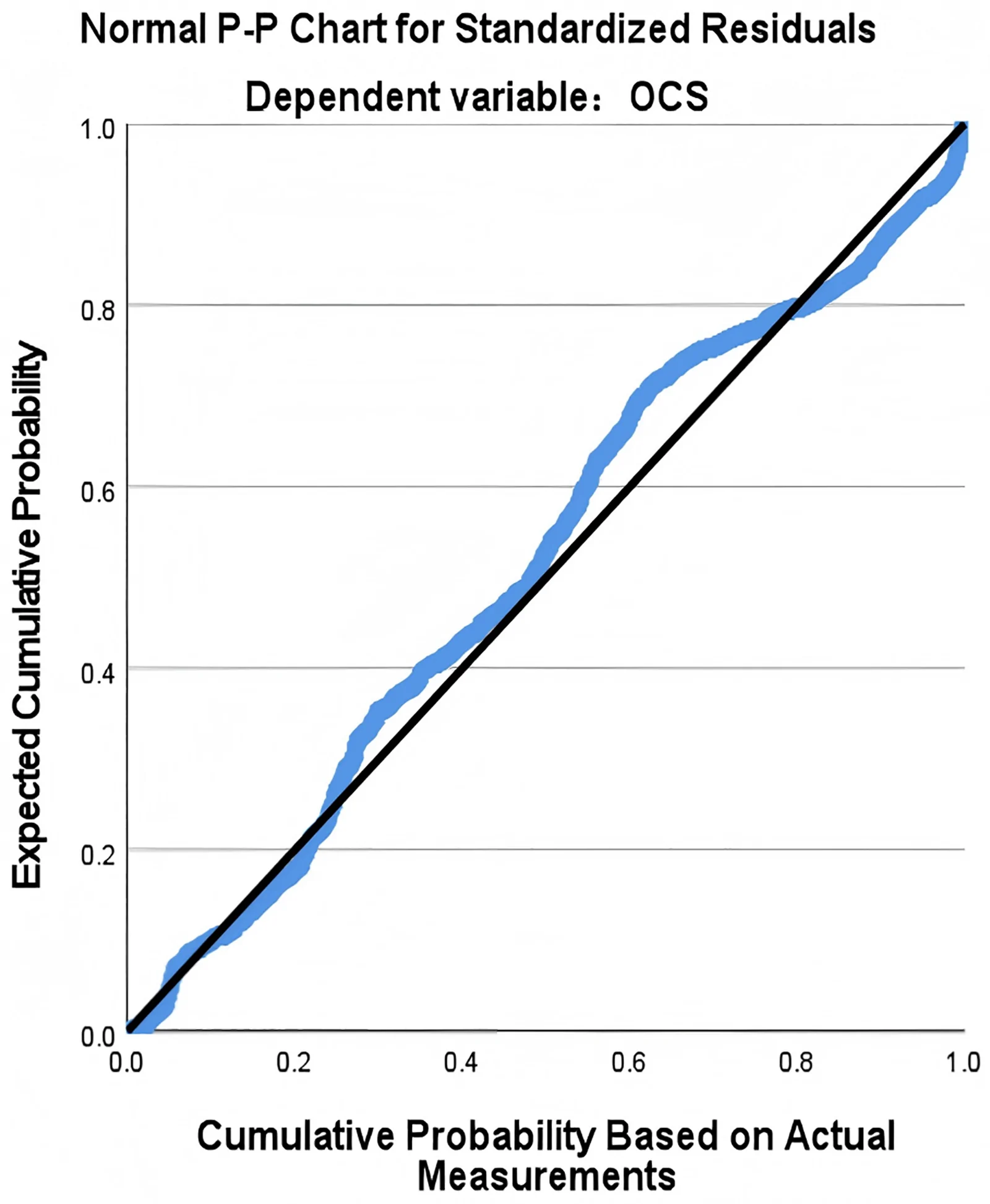
P-P Chart. Note: The observed cumulative probabilities closely follow the expected cumulative probabilities along the diagonal line, confirming that the residuals are normally distributed and that the regression model assumptions are satisfied.

Furthermore, as shown in Fig 2 the histogram of standardized residuals exhibits a symmetric distribution centered around zero, approximating a normal distribution, thereby satisfying the normality assumption of regression analysis. In Fig 3, the normal P-P Chart of standardized residuals shows that the observed cumulative probability is closely clustered around the diagonal line representing the expected cumulative probability, further validating the normality of the residuals. This satisfies the conditions of homoscedasticity and independence of the data.

## 5. Discussion

This study employs a cross-sectional correlational design to examine the relationship between the six design attributes of the PETE curriculum and overall satisfaction, as well as differences in satisfaction levels by major, gender, and grade level. The following discussion is based strictly on the results of descriptive statistics, analysis of variance, correlation analysis, and multiple regression analysis, and provides theoretical interpretations in light of the educational and cultural contexts of China and Guangdong Province.

Analysis of variance revealed no significant differences in satisfaction with the PETE program by gender or grade level, indicating that the program's design and implementation are equitable across demographic groups. This is consistent with the findings of Werfhorst et al., who concluded that standardized curricula narrow gaps between groups through goal-oriented teaching methods [47]. Notably, these findings challenge the Western assumption that female students experience higher career-related stress in PETE programs [48]. For example, in Spain's Physical Education and Sports Teaching (PESTE) program, female students account for only 20% [49]; this may be one reason why university applicants describe the PESTE program as “highly masculinized” [50]. This long-standing underrepresentation, combined with authoritarian teaching attitudes, stigmatizing behaviors toward female students, and traditional teaching methods targeting female students, constitutes key factors that undermine female students’ participation in sports [51]. However, in the physical education context of China and Guangdong Province, the following factors may have mitigated gender disparities. First, a competency-oriented assessment culture. Physical education courses in Guangdong's higher education institutions emphasize athletic ability as the core assessment criterion rather than the performance of gender roles; both practical exams and teaching skills competitions are evaluated using the same standards, thereby reducing the direct impact of traditional gender norms on the learning experience [20,42]. Second, gender balance in the job market. According to recent physical education teacher recruitment announcements in Guangdong Province, the male-to-female ratio for these positions is generally balanced. Recruitment prioritizes professional skills and teaching ability as the primary selection criteria, with no apparent gender bias, thereby reducing female students’ career-related anxiety. Third, an atmosphere of collaborative learning within a collectivist culture.

Although no significant differences emerged by gender or academic year, significant between-group differences were observed between PE and ST majors. ST majors reported lower satisfaction with course objectives (p < 0.05), knowledge acquisition (p < 0.05), and competence development (p < 0.05), potentially due to curricula prioritizing teaching competence over sport-specific competencies. Their career orientation of this group was more toward a coaching domain sport management orientation, resulting in a mismatch between the curriculum and career trajectories.Ojeda-Nahuelcura demonstrated that PE majors derived greater benefits from active learning modules than ST majors [52]. Fieldwork experiences diverged significantly due to curricular disparities, PE majors perceived content as directly relevant to specialization and careers, whereas ST majors deemed active learning components tangential to their aspirations. Meanwhile, chi-squared analysis further highlighted the problem: ST majors were significantly less willing to pursue a teaching career than PE majors (χ² = 24.918,p = 0.002).From the perspective of self-determination theory, this difference may stem from variations in the degree to which autonomy needs are met. Autonomy refers to an individual's perception that their behavior stems from their own will and that the goals of their activities align with their personal values [53]. The educational objectives of the physical education program align closely with the curriculum design; from the moment they enroll, students clearly set their career path toward becoming physical education teachers in elementary and secondary schools, and the course content directly serves this goal. Consequently, their need for autonomy is satisfied to a high degree. In contrast, the career pathways for students in the Sports Training program are more diverse, including roles such as coaches, sports administrators, strength and conditioning coaches, and entrepreneurs in the sports industry. However, both in Guangdong Province and nationwide, the curriculum for the Sports Training program remains primarily focused on teacher training, lacking alternative modules such as coaching leadership, sports management, sports team operations, and event organization. This curriculum design results in the autonomy needs of Sports Training students not being fully met [54]. The policy and industrial context in Guangdong Province has further amplified this disparity. The province's reforms to physical education class hours and the expansion of teacher recruitment have provided students in physical education programs with clear employment prospects, thereby strengthening their sense of competence and professional identity. Gong et al. (2023) point out that professional identity mediates the relationship between coursework and career outcomes [55]. Physical education majors gradually internalize the identity of “prospective teachers” through their coursework, creating a positive cycle between their course satisfaction and willingness to teach; in contrast, sports training majors face an ambiguous sense of identity—neither purely “coaches” nor typical “teachers.” The curriculum fails to provide them with a clear path for constructing a professional identity, leading students to question the value of the program and, consequently, reducing their satisfaction and willingness to teach.

Correlation analysis revealed competence development as the core determinant of student satisfaction in PETE curricula, with knowledge acquisition (r = 0.808) and competence development (r = 0.803) showing the strongest association with overall satisfaction. This aligns with Baharin and Hanafi proved that specialized skill utility enhances career readiness [56]. Specifically, students with theoretical knowledge of student psychology and curriculum design, as well as practical skills such as movement modeling and instructional design, were more satisfied because these combined skills fit the multifaceted needs of the contemporary physical education teaching profession. This finding is consistent with the predictions of Self-Determination Theory (SDT): a sense of competence is the primary psychological need driving satisfaction [26]. In the context of the PETE curriculum, students are most concerned with whether they can acquire transferable professional knowledge and practical teaching skills through the course. From a broader perspective, the number of physical education class hours in primary and secondary schools and the scale of physical education teacher recruitment have expanded significantly, leading to a stronger demand among students for “practical knowledge” and “practical skills.” The high correlation with knowledge acquisition reflects students’ emphasis on the alignment between course content and teacher certification exams as well as civil service recruitment; the high correlation with competence development indicates that students are highly sensitive to the quality of practical components such as microteaching and physical education fieldwork. In contrast, the correlations with course structure and course content are weaker, suggesting that when evaluating a course, students place greater importance on “what they have learned” and “what they can do” rather than on the course's organizational format or scope. However, Low scores on innovative content (Q16: M = 3.48) and Practical application (Q9: M = 3.47) expose systemic flaws. Insufficient training in student-adapted instruction and emerging pedagogies such as sports technology may weaken students’ readiness to deal with practical challenges,a critique echoed by Cox et al regarding digital competence deficits in teacher education [57].

Contributions to Theory and Practice. Based on SDT, this study constructed a direct effects model of PETE course satisfaction. The results of correlation and regression analyses partially supported this model: knowledge acquisition and skill development had the strongest predictive power for overall satisfaction (H1b supported); significant differences were found across major types in terms of course objectives, knowledge acquisition, skill development, and overall satisfaction (H2a and H2e supported); and no significant differences were observed for gender or grade level (H3a and H3b supported). The theoretical contributions of this study are as follows: First, it provides empirical data on PETE course satisfaction in Guangdong Province, China; second, it identifies major type as a key grouping variable; third, it proposes operationalized course dimensions based on SDT.

Based on the above findings and in conjunction with the three basic psychological needs of SDT, the following practical optimization strategies are proposed.

First, to address the lack of autonomy among students majoring in sports training, a modular course design should be adopted. Additional elective courses, such as coaching leadership and sports management, should be introduced, and a practice community linking the university and the community should be established. This grants students the freedom to choose learning paths based on their career aspirations, directly supporting their need for autonomy.

Second, to address the need for competence, the gap between theory and practice should be bridged. Through collaboration with school districts and community sports programs, service-learning should be integrated to enable students to apply their knowledge in real teaching contexts. A reflective supervision mechanism is implemented to help students validate and consolidate their professional skills through practice, thereby enhancing their sense of competence.

Third, to address the issue of cognitive load, a progressive cognitive scaffolding system is implemented. In complex courses such as exercise physiology, a structured scaffolding model is adopted, prerequisite knowledge maps are developed, and a spiral curriculum design is employed. This ensures that students master knowledge step by step, preventing frustration regarding their sense of competence.

Fourth, to address the need for a sense of belonging, we reform assessment methods. We adopt formative and value-added assessment approaches, introducing video-based peer assessment and reflective portfolios. Through peer feedback and community-based learning, we strengthen connections and mutual support among students, thereby fulfilling their need for a sense of belonging.

## 6. Limitations and future Research

### 6.1 Limitations

This study has the following three limitations. First, measurement bias and design limitations. Self-reported data may be influenced by social desirability bias, leading to an overestimation of satisfaction scores; the limited number of items (two) in the evaluation and assessment dimensions resulted in low reliability, which weakened the construct discriminant validity. Second, limitations regarding sample representativeness. The sample was limited to universities in Guangdong Province, a region with high demand for physical education teachers, which may have led to an overestimation of overall satisfaction; therefore, caution is warranted when generalizing the conclusions to central and western provinces and other professional groups. Third, statistical analysis was insufficient. This study did not use structural equation modeling to test for mediating or moderating effects. In summary, the conclusions of this study should be regarded as exploratory findings, and the proposed curriculum optimization framework requires further validation through longitudinal designs, standardized SDT scales, and structural equation modeling.

### 6.2 Future research

Cross-Regional Comparison and Geospatial Analysis of PETE course Effectiveness. An in-depth comparative analysis of PETE courses in eastern, central, and western provinces of China to expand the sample size across regions helps to reveal how socioeconomic context moderates curriculum satisfaction.

Longitudinal tracking design with longitudinal satisfaction to monitor career trajectories. Adopting a longitudinal research design to correlate student satisfaction with post-graduation indicators such as career retention, job performance, and burnout trajectories can deepen understanding of the long-term impact of the course and analyze the relationship between satisfaction and burnout.

Qualitative in-depth, phenomenological study of teaching resistance. By conducting in-depth interviews with sports training majors who exhibit “teaching resistance,” including salary expectations, social perceptions of sports training careers, or misplaced course identity, the underlying causes can uncover root causes beyond quantitative data.

### Informed consent statement

This study obtained participants’ consent online: members of the research team provided the Informed Consent Form and related explanations to eligible third- and fourth-year undergraduate students (who have completed all required credits for physical education teacher education courses), participants read the form independently and decided whether to sign it, then signed their names in the signature section at the end of the form to confirm their consent to participate in the study; the informed consent was obtained after the ethics review committee's approval and before participants completed the questionnaire, with the specific date based on the participant's signature. The consent obtained covers participants agreeing to participate in the questionnaire survey phase of this study and complete the 42-question questionnaire according to the survey procedures, agreeing that their questionnaire responses may be used for academic analysis and presentation of research findings in this study, and agreeing that the research team may use relevant data while protecting their privacy (with explicit understanding that research results will not include personally identifiable information). As a questionnaire survey-type non-interventional research study, all participants were fully informed of the following: anonymity (research results will not contain personally identifiable information, only the research team has access to original information, and personal information is kept confidential), research purpose (examining the current status of physical education teacher education course implementation from the student satisfaction perspective to provide a basis for course optimization), data usage (responses are used solely for academic analysis in this study to support developing a quality optimization framework for physical education teacher education courses), and participation risks (the survey has low risks, with no physical/mental harm or rights infringement); meanwhile, the informed consent process strictly adhered to the World Health Organization (WHO) guidelines on informed consent, ensuring voluntariness, adequate information, and the right to withdraw at any time without penalty. Additionally, the study's participants are third- or fourth-year university undergraduates, all adults with full legal capacity who do not belong to vulnerable groups or minors, so there is no need to obtain consent from their legal guardians, and participants can independently decide whether to participate in the study and sign the informed consent form.

### Biographical note

Yaqin Ren, Associate Professor, Ph.D., Graduate supervisor (Master's level), Research interests: School physical education, teacher education.

Taoqing Liu, Master's degree candidate, Research interests: School physical education, teachers’ digital Literacy.

## Supporting information

S1 File Raw Data (XLSX)
